# Supplementary material for: Molecular Mechanisms of Chlorophyll Deficiency in Ilex × attenuata ‘Sunny Foster’ Mutant
Source: Plants (Basel). 2024 May 7;13(10):1284. doi: 10.3390/plants13101284 (PMC11124982; doi:10.3390/plants13101284)
Supplement: Supplementary file 1 [file plants-13-01284-s001.zip › Supplementary Figures (S1-S3).pdf]

Supplementary Figure Legends

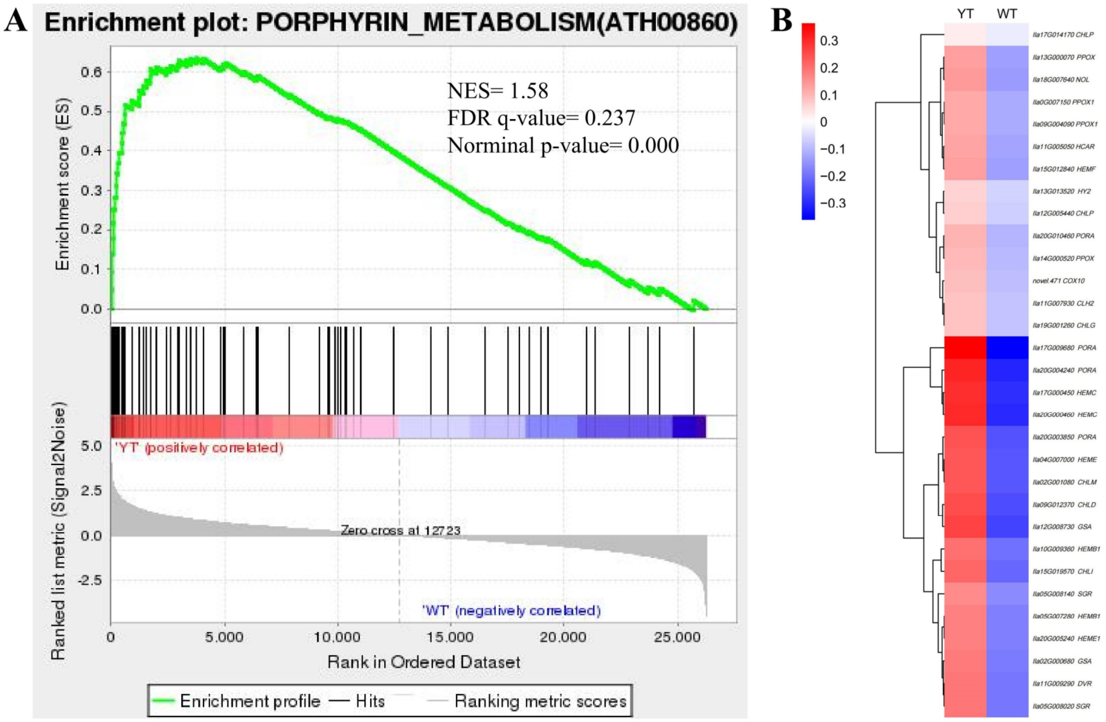

Figure S1 GSEA analysis based on KEGG data in porphyrin metabolism in YT. (A) GSEA enrichment plot in porphyrin metabolism pathway. (B) Hierarchical cluster analysis of GSEA enriched genes in porphyrin metabolism pathway. Red indicates up-regulated genes, and green indicates down-regulated genes. YT: mutant *I.* × *attenuata* ‘Sunny Foster.’

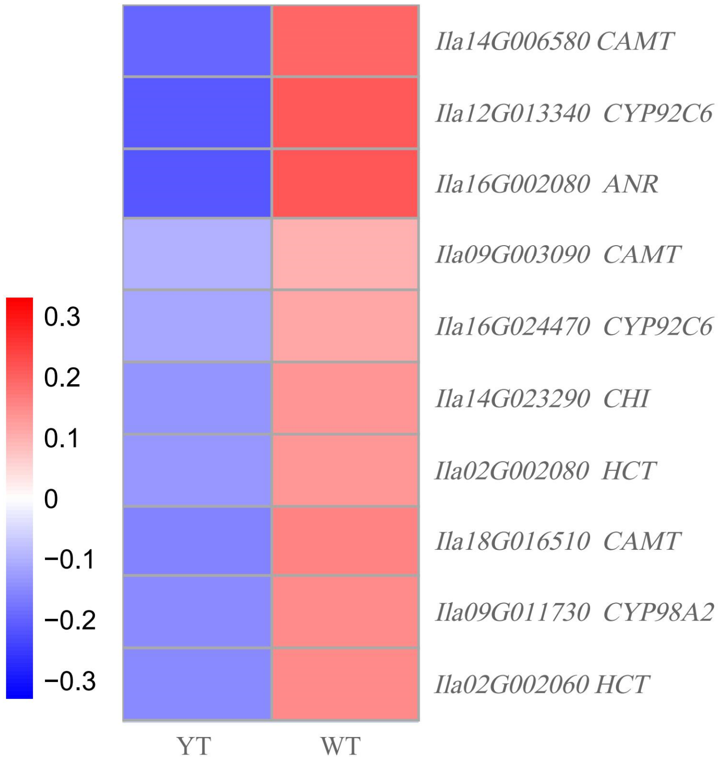

Figure S2 Heatmap of DEGs involved in flavonoid biosynthesis between YT and WT. The mean $\log_2$ (Fold Change) values for the DEGs were calculated from three biological replicates for YT and WT. Red indicates up-regulation, and blue indicates down-regulation. WT–wild-type *Ilex* × *attenuata* ‘Foster #2’; YT–mutant *I.* × *attenuata* ‘Sunny Foster.’

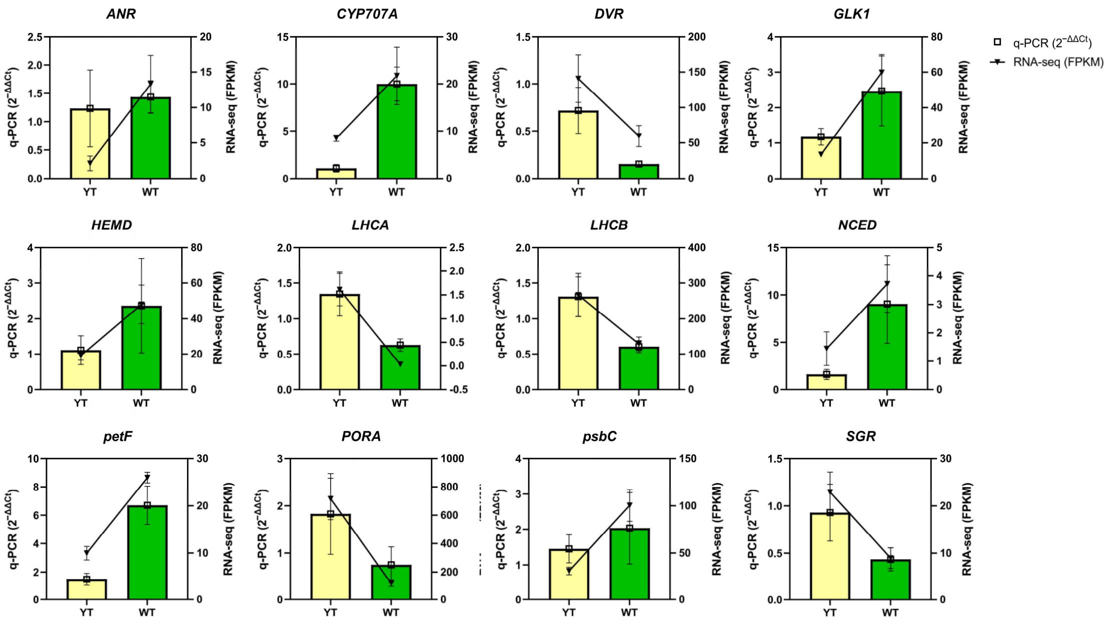

Figure S3 The expression levels of 12 genes in RNA-seq and qRT-PCR.

WT–wild-type *Ilex* × *attenuata* ‘Foster #2’; YT–mutant *I.* × *attenuata* ‘Sunny Foster.’
