# Supplementary material for: Molecular Mechanisms of Chlorophyll Deficiency in Ilex × attenuata ‘Sunny Foster’ Mutant
Source: Plants (Basel). 2024 May 7;13(10):1284. doi: 10.3390/plants13101284 (PMC11124982; doi:10.3390/plants13101284)
Supplement: Supplementary file 1 [file plants-13-01284-s001.zip › Supplementary Tables (S1, S2, S9).pdf]

Supplementary Tables

Table S1 Comparative analysis of epidermal features of YT and WT.

Histological parameters	YT	WT
Stomata length (um)	33.65±1.76a	35.06±0.73a
Stomata width (um)	31.08±2.52a	28.67±1.52a
Stomata density (per um^2)	317.70±13.30b	358.14±5.79a
Closed stomata density (per um^2)	240.55±5.30a	5.14±0.72b

Note: The values represented the mean±SD. Different lowercase letters indicate significant differences at $P<0.05$.

Table S2 Summary of the sequencing and mapping results between YT and WT.

Samples	Total reads	Clean reads	Q20	Q30	GC Content	Mapping rate
YT-1	44,070,410	42,578,268	96.77%	91.42%	44.33%	68.08%
YT-2	47,481,470	46,073,680	96.80%	91.39%	44.08%	67.57%
YT-3	45,437,032	44,412,792	96.94%	91.75%	44.30%	67.94%
WT-1	43,600,624	42,072,156	96.98%	91.88%	44.52%	67.44%
WT-2	43,523,698	42,160,692	97.17%	92.24%	44.49%	68.32%
WT-3	44,402,320	43,096,068	96.87%	91.65%	44.37%	67.81%
Total	268,515,554	260,393,656	96.98%	91.88%	44.35%	67.86%

Table S9 List of primers used for qRT-PCR analysis.

Gene ID	Forward primer	Reverse primer
GLK1(*Ila20G011400*)	ATGAGCCTTTGGGTAGCACC	GTCTGCTGGTGGTGGATGAA
PORA(*Ila17G009680*)	CCCATTGTTCAGGGCTCTGT	CACCTTCCTGGCCTTCTCTG
HEMD(*Ila09G014230*)	ACTGGTATTTGGAAGGGGCG	TGAATCGGTGTCAGCTGCAT
SGR(*Ila05G008020*)	ATTGTTATGTGAGCGGGCCA	TTAGTGGCCCCCAACATTCC
NCED1(*Ila20G005950*)	CCAGTGTCCAAATTGCAGGC	GTAGCTTGCTGACCCGTTCT
CYP707A1(*Ila10G003260*)	TTCAATGGGGTGGCCTCTTG	GCCTGAACCAGCTTCCTCAT
ANR(*Ila16G002080*)	TTCAATGCCCCCATAGCAGG	TCGCCTGAGCTGATCGTTAC
LHCA(*Ila16G017490*)	CATTGCTTCCACTGCGCTTT	TTGCCCATCTGCCATGGATT
PsbC(*Ila0G015340*)	GGCCGGAGCAATGAACCTAT	GTCTCAGGTCCCAGAAGTGC
LHCB(*Ila15G017050*)	CTTGGGGCTCTTGGATGTGT	CCAATGGGTCGAAGCTTCCT
petF(*Ila17G015180*)	GGCGAAGAGACCGAGTTTGA	GCAAGTGAGCAGGTAACCCT
DVR(*Ila11G009290*)	TGTCTCTTGCCTTGCTAGCC	CCTGTCCTCCCAAGCTCTTG
Actin	CCACCTACAACTCCATCAT	TTCCTTGCTCATACGATCA
